# One-dioptre toric IOL versus spherical IOL in eyes with low preoperative corneal astigmatism

**DOI:** 10.1007/s10792-022-02571-4

**Published:** 2022-11-23

**Authors:** Carlo Bellucci, Angela Panico, Salvatore A. Tedesco, Arturo Carta, Stefano Gandolfi, Roberto Bellucci, Paolo Mora

**Affiliations:** 1 Ophthalmology Unit, Department of Medicine and Surgery, University of Parma - Via Gramsci 14, 43126 Parma, Italy; 2 San Giuseppe E Melorio Hospital, Santa Maria Capua Vetere, Italy; 3 Vista Vision Surgical Centre, Verona, Italy

**Keywords:** Cataract surgery, Pseudophakia, Astigmatism, Toric IOL, Spherical IOL

## Abstract

**Purpose:**

To investigate the advantages/disadvantages of a 1.0 D toric IOL vs spherical IOL after regular phacoemulsification in eyes with preoperative astigmatism ≤ 1 D.

**Methods:**

Retrospective comparative series involving pseudophakic eyes with preoperative topographic astigmatism ≤ 1.0 D implanted either with monofocal 1.0 D Toric IOL (T-group), or with spherical IOL (S-group). The postoperative refractive astigmatism (PRA, i.e. surgically induced + corneal) was the main outcome; also considered in the analyses were the uncorrected and best-corrected distance visual acuity (VA). The data were referred to the last postoperative follow-up visit, 2 to 4 months after surgery.

**Results:**

A total of 60 eyes were included: 30 in the T-group and 30 in the S-group, matched for patient’s age, laterality, and axial length. Before surgery, the mean corneal astigmatism was 0.62 ± 0.39 D in the T-group and 0.54 ± 0.33 D in the S-group (*p* = 0.4). In the S-group, PRA was 0.73 ± 0.37 D, higher than the corresponding preoperative corneal astigmatism (*p* = 0.040). In the T-group, PRA was 0.58 ± 0.31 D; the variation was not statistically significant. Uncorrected VA was significantly better in the T-group vs the S-group (*p* = 0.007), and the best-corrected VA was comparable in the two groups.

**Conclusion:**

The present study indicated that in eyes with very low preoperative astigmatism, 1.0 D toric IOLs were able to limit the increase of the PRA instead of those observed with the spherical IOLs. This could support the better uncorrected VA recorded in the T-group.

## Introduction

Eyes addressed to cataract surgery with monofocal intraocular lens (IOL) implants presenting corneal astigmatism ≤ 1.0 D usually receive spherical IOL. However, the refractive outcome of these eyes with preoperative astigmatism within a physiological interval can be worse than the outcome of eyes presenting higher astigmatism but implanted with toric IOLs [1–3]. The against-the-rule or the oblique anterior corneal astigmatism, the posterior corneal astigmatism and the surgically induced astigmatism (SIA) are the main contributors to this residual refractive error in eyes receiving spherical IOL [4–6].

One-dioptre toric monofocal IOLs correct about 0.60–0.70 D of astigmatism at the spectacle plane, depending on the “A” constant [7, 8]. Even in eyes with an almost spherical cornea, these toric implants might contribute to a final refractive astigmatism lower than that of spherical IOLs [3]. To deeper investigate this issue, we compared the refractive results of two groups of eyes with low preoperative corneal astigmatism implanted either with toric or with spherical IOLs.

## Materials and methods

Regular pseudophakic patients implanted with monofocal IOL between January 2019 and December 2020 were considered for this retrospective comparative study, which followed the principals of the declaration of Helsinki and was approved by the Area Vasta Emilia Nord Etic Committee (#126/2022). Inclusion criteria were preoperative corneal astigmatism (simulated keratometry in the 3-mm central cornea, SIM K) ≤ 1.0 D; regular corneal topography; targeted IOL power between 18.0 D and 25.0 D as calculated with the Kane formula [9]; uneventful in-the bag IOL implantation; no combined ocular surgery; final best-corrected visual acuity (BCVA) ≤ 0.1 LogMAR.

The eyes included had received either a 1.0 D toric single-piece monofocal IOL (PerfecTor, Hanita Lenses, Israel) following temporal clear cornea 2.2 mm incision; or a spherical single-piece monofocal IOL (Incise, Bausch & Lomb, USA) following 2.2 mm incision on the steepest axis when the corneal astigmatism was > 0.5, and horizontally for corneal astigmatism ≤ 0.5 D. The incisions are known to produce about 0.25 D of SIA [10, 11].

Preoperatively, the corneal astigmatism was assessed by the Sirius Scheimpflug camera topographer (CSO, Italy), with the refractive index set at 1.3375. Three measurements were taken for each eye, and the mean values of the Sim K were noted in terms of power (dioptres) and axis (degrees). The direction of the measured astigmatism was assumed as follows: with-the-rule (WTR): 60°–120°; against-the-rule (ATR): 0°–30° and 150°–180°; oblique (OBL): 30°–60° and 120°–150°. The axial length was measured with the IOL Master 500 Optical Biometer (Zeiss, Germany).

All surgeries were performed by the same expert surgeon (PM), the Stellaris phacoemulsifier with the 1.8 microincision phaco tip (Bausch & Lomb, Rochester, USA) was used, with the Medicel 1.8 mm injector used for all implantations (Medicel AG, Switzerland). For the spherical monofocal implants, the Barrett Universal II formula with an emmetropic target was adopted. The 1.0 D toric IOLs were aligned along the axis suggested by the Kane Toric formula using a Mendez ring with reference to the horizontal corneal meridian marked at the slit lamp in the operating theatre. An emmetropic target was also aimed for toric implants.

Data for the study refer to the last postoperative follow-up visit, which occurred for all cases 2 to 4 months after surgery. At that time, corneal topography was performed following the same procedure adopted preoperatively. Automated refraction (Topcon KR800) was employed to measure the objective refraction (as the mean of 3 measurements), refined by the Jackson cross-cylinder to assess the BCVA (early treatment diabetic retinopathy—ETDRS—chart at 4 m). The visit considered for the study had to report the IOP value and the details of the slit-lamp examination of the anterior segment, vitreous (by retroillumination), and posterior pole (under mydriasis using a 90-dioptre lens). For toric IOLs, the proper alignment with the preoperative target was checked during the mydriatic slit-lamp evaluation.

### Statistical analysis

The sample size was calculated considering the null hypothesis rejection for ≥ 0.25 D difference in the postoperative refractive astigmatism, 95% confidence limits and 0.30 D as the standard deviation of the measurements. This calculation showed that at least 23 eyes per group should be considered for binary comparison purposes. Means and standard deviations were reported for continuous variables with a normal distribution. To analyse data referring to eyes implanted with Toric IOL (T-group) and eyes implanted with Spherical IOL (S-group), the Student’s t or the Fisher’s exact tests were used for the normally distributed parameters and the corresponding nonparametric tests for the non-normal distributions. Statistical analyses were performed using commercial software (SPSS version 25.0; IBM, Armonk, NY, USA); the P < 0.05 was considered significant.

The online calculator tools of the American Society of Cataract and Refractive Surgery (available at www.ascrs.org) were used to represent the corneal astigmatism before and after surgery, the SIA, and the pseudophakic clinical astigmatism [12–14]. The internal astigmatism in the operated eyes was assessed with the online ASSORT Vector calculator of the International Refractive Surgery Society (available at www.isrs.org) and computed as the difference between the corneal astigmatism and the clinical astigmatism [15].

## Results

Sixty eyes of as many patients were included in the study: 30 in the T-group and 30 in the S-group. The study groups were matched for patient’s age, eye laterality and axial length. Table 1 shows the preoperative characteristics of the two groups, statistically comparable for all the considered parameters except for the IOL power, due to the different “A” constant of the two models. Incision location in the S-group was ATR in 14 eyes, WTR in 10 eyes, OBL in 6 eyes. Intraoperative IOL alignment in the T-group was ATR in 19 eyes, WTR in 6 eyes, and OBL in 5 eyes. At the final postoperative examination, 21 Toric IOLs were found aligned within 5° from the intended axis; 7 were found misaligned by 6°–10°, and 2 were misaligned by 11° to 16°. The mean misalignment was 4.2 ± 2.7°. The uncorrected distance visual acuity was 0.08 ± 0.07 LogMAR in the T-group and 0.13 ± 0.07 LogMAR in the S-group (*p* =  0.007). The BCVA was comparable in the two groups, at 0.01 ± 0.04 LogMAR and 0.02 ± 0.04 LogMAR, respectively.

**Table 1:** Characteristics of the eyes and implants in the two study groups

Parameter	Unit	1-D Toric IOL	Spherical IOL	P
No. of eyes	n	30	30	
Patient age	y	77.1 ± 6.2	78.4 ± 6.3	0.423
Male/Female	n	13/17	13/17	1.000
Right/Left eye	n	14/16	16/14	0.976
Axial length	mm	23.3 ± 1.1	23.2 ± 1.2	0.737
“A” constant	n	117.7	119.2	
IOL power	D	20.43 ± 1.44	21.75 ± 1.25	0.001
Toric IOL alignment	WTR/ATR/OBL	6/19/5	–	
Preoperative corneal astigmatism (mean SD)	D	0.62 ± 0.39	0.54 ± 0.33	0.394
Postoperative corneal astigmatism (mean SD)	D	0.56 ± 0.38	0.55 ± 0.39	0.94

*WTR* with-the-rule; *ATR* against-the-rule; *OBL* oblique

The uncorrected near visual acuity was also tested to check if the 1.0 toric implant could play a role in near vision for these eyes founding no difference between the two groups.

**Fig. 1:**
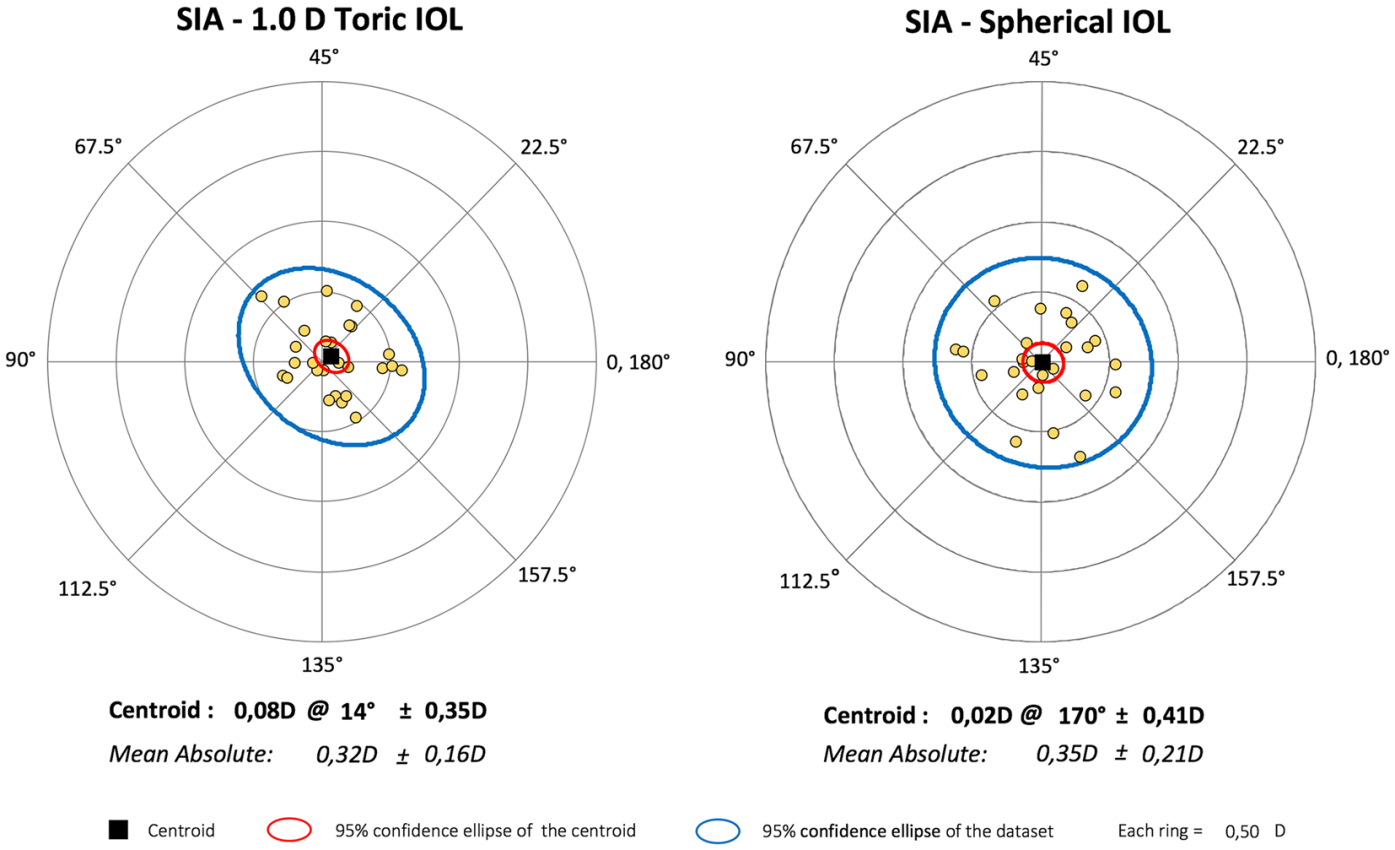
Surgically induced astigmatism (SIA) in the two study groups

Before surgery, the mean corneal astigmatism was 0.62 ± 0.39 D in the T-group and 0.54 ± 0.33 D in the S-group. At the final evaluation, it was 0.56 ± 0.38 D and 0.55 ± 0.39 D, respectively, without significant changes in both the inter- and intragroup comparison. Mean SIA was 0.32 ± 0.16 D in the T-group and 0.35 ± 0.21 D in the S-group (Fig. 1).

The postoperative clinical refractive outcome is detailed in Table 2, with a positive notation for the cylinder component. Although the mean clinical refractive astigmatism was slightly higher in the S-group, this difference, as well as that referred to the spherical component of the two groups, did not test significantly.

**Table 2:** Postoperative clinical refraction at the spectacle plane

Postoperative refraction	1.0 D Toric IOL	Spherical IOL
Sphere	−0.58 ± 0.43 D	−0.58 ± 0.82 D
Cylinder	+ 0.58 ± 0.32 D	+ 0.73 ± 0.37 D
Cylinder axis	79.4° ± 70.4°	64.8° ± 62.9°

**Fig. 2:**
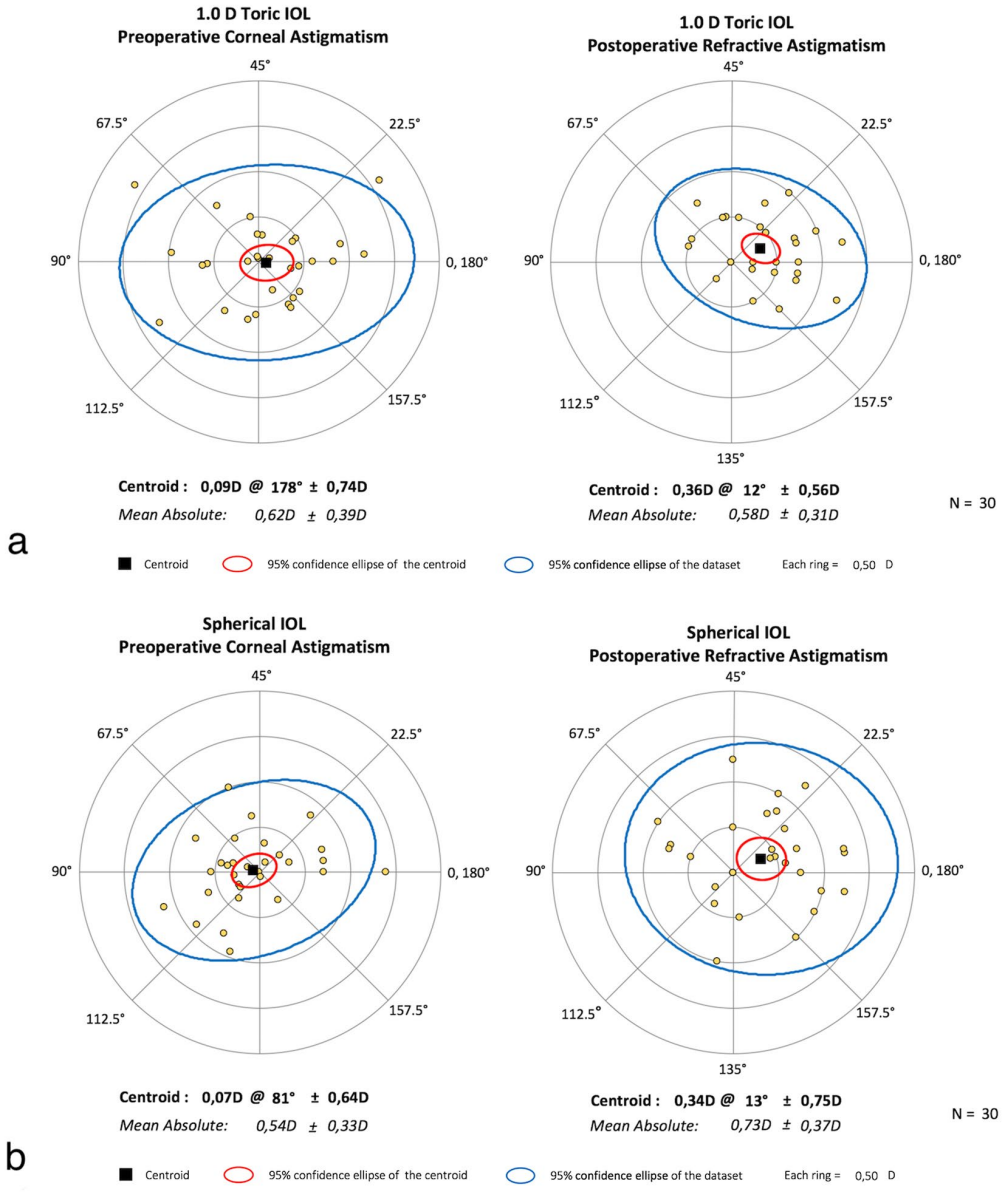
**a** and **b** Preoperative corneal astigmatism (left side) and postoperative refractive astigmatism at the spectacle plane (right side) in the two groups (a: toric; b: spherical)

Figure 2a and b reports the preoperative *corneal* astigmatism (left side) and the postoperative *refractive* astigmatism (PRA) at the spectacle plane (right side) for the two groups. There was no statistical difference between the two groups in the postoperative refractive outcomes referring to both the amount of the PRA and the corresponding centroids. However, in the S-group the PRA (i.e. SIA + corneal components) was 0.73 ± 0.37 D (Fig. 2b), significantly higher (*p* = 0.040) than the corresponding preoperative corneal astigmatism, i.e. avoiding the lens and surgical induced components. In the T-group, the mean PRA was 0.58 ± 0.31 D (Fig. 2a); the variation with respect to the corresponding preoperative assessments was not statistically significant.

**Fig. 3:**
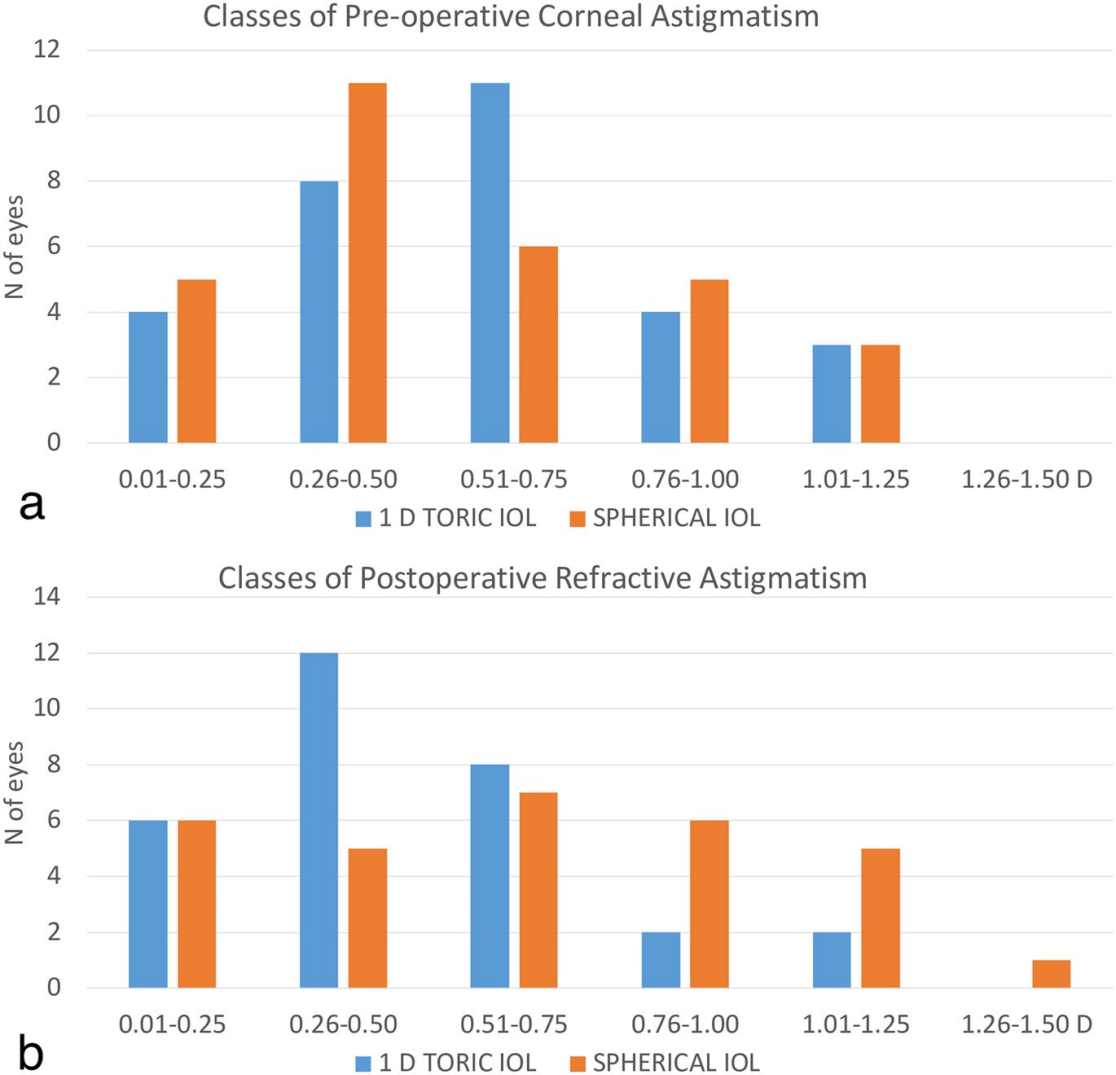
**a** and **b** Amounts of preoperative astigmatism (part a) and postoperative refractive astigmatism (pat b) in the two study groups

The overall distribution of the PRA is shown in Fig. 3b. The number of eyes with < 0.75 D of PRA was 26 in the T-group and 18 in the S-group (p = 0.039); 2 eyes in the T-group and 6 eyes in the S-group had PRA > 1.0 D. Twelve eyes in the T-group and 16 eyes in the S-group had preoperative corneal astigmatism ≤ 0.5 D (Fig. 3a). Considering these selected subgroups, the mean PRA was 0.50 ± 0.34 D in the toric implanted eyes and 0.64 ± 0.42 D in the eyes with the spherical IOL. While the inter-subgroups difference (T vs S subgroup) was not statistically significant, the intra-subgroup difference tested significant in the sole spherical implanted eyes (*p* = 0.003).

**Fig. 4:**
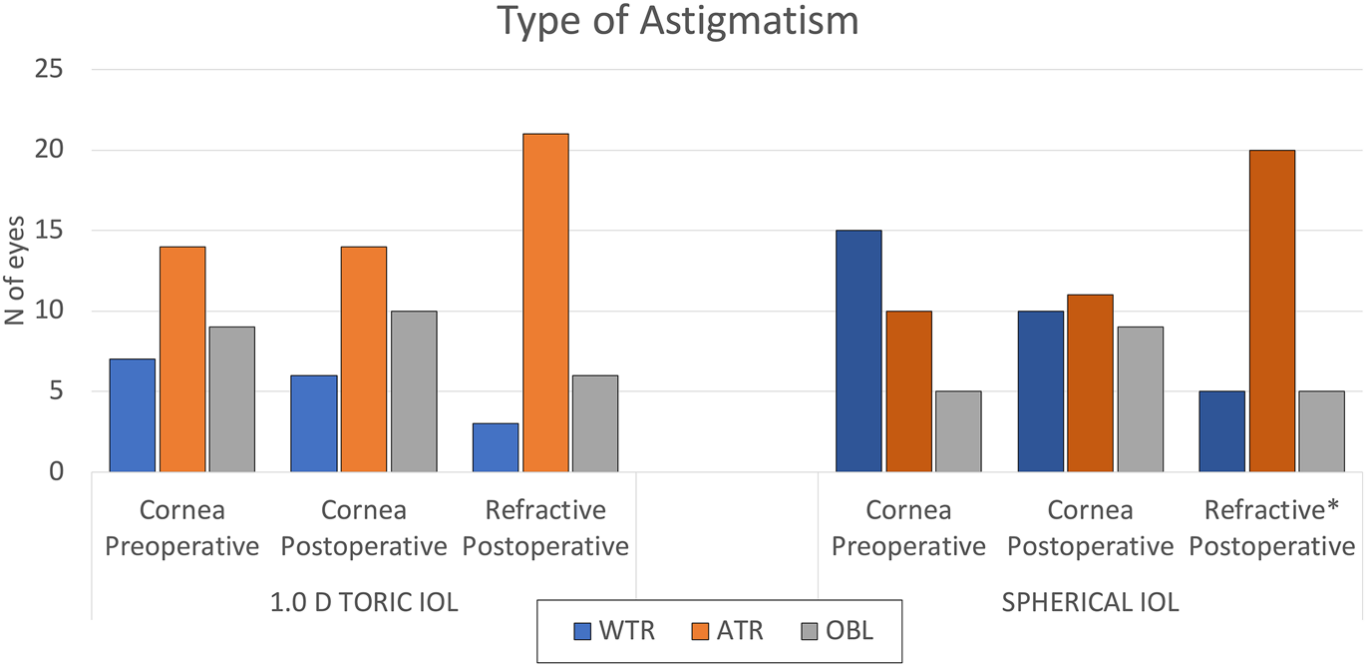
Orientation of the preoperative and postoperative corneal and refractive astigmatism in the two study groups. *WTR* with-the-rule; *ATR* against-the-rule; *OBL* oblique astigmatism

The axis of the preoperative corneal astigmatism and that of the PRA are reported in Fig. 4. In the postoperative assessment, ATR refractive astigmatism was predominant in both groups. The S-group showed a significant axis shift from the preoperative corneal values to the postoperative refractive values (*p* = 0.016).

The mean internal astigmatism in the pseudophakia was 0.73 ± 0.43 D in the T-group and 0.70 ± 0.33 D in the S-group. There was no difference between groups in the magnitude and in the axis of the internal astigmatism as calculated with the adopted method.

## Discussion

The purpose of this study was to evaluate if the implant of 1.0 D Toric monofocal IOLs offered any advantage or disadvantage over spherical IOLs in eyes with preoperative topographic astigmatism ≤ 1.0 D, which conventionally represent the target for spherical IOL implants.

For the comparison, we arranged that the preoperative topographic astigmatism was similar in the two study groups and that the intraoperative and postoperative characteristics had an uneventful run. The low and comparable data of SIA (as shown in Fig. 1) and the unsignificant variation of the topographic astigmatism between the pre- and postoperative evaluation in both groups confirmed the respect of these conditions. Given the loss of the influence of the toricity of the natural lens after surgery, we consider the PRA as the main functional outcome.

Although averagely higher in the S-group (0.73 ± 0.37 D vs 0.58 ± 0.31 D in the T-group), PRA was statistically comparable in the two groups. This lack of significance may rely on several factors: the lower toricity of the 1.0 D toric IOLs at the spectacle plane, the reduction of the corrective effect induced by the intra- or postoperative misalignment [16, 17], the potential effects of decentration and tilt [18, 19] and the variations in the posterior corneal astigmatism [20]. All these variables typically have little influence on the total refractive astigmatism in pseudophakia, but they may become relevant when the corneal contribution is very low [21–23]. Moreover, the total amount of the pseudophakic refractive astigmatism cannot be explained by the sole refractive assessment of the anterior segment, even if it is conducted in detail [24].

Some outcomes were, however, in favour of the use of the toric IOL.

1. The difference between the preoperative corneal astigmatisms and the corresponding PRA was only significant in the S-group; this difference tested even more significant when considering the 16 eyes in the S-group with the lowest preoperative corneal astigmatism (≤ 0.5 D). The finding may indicate that the compensative toric component of the natural lens was not entirely substituted by the spherical IOL. This also considering the low and comparable SIA and the significant axis shift from the preoperative corneal values to the postoperative refractive values in the S-group.
2. The uncorrected distance visual acuity was significantly better in the T-group; this evidence too was possibly related to a better compensation of the corneal astigmatism by the toric IOL. There was no difference between the two groups for the uncorrected near vision, meaning that although the 1.0 toric implant did not improve uncorrected near vision in our patients, nor it compromised it in any case.
3. The number of eyes resulted with PRA < 0.75 D was significantly lower in the S-group.

In the literature, the correction of low corneal astigmatism with toric IOLs is reported to provide satisfactory results. Ernest and Potvin demonstrated a better refractive outcome with the 1.5 D toric IOL in eyes with 1.06 D of mean corneal astigmatism [4]. The mean PRA was 0.31 D in the toric group and 1.06 D in the spherical group (P < 0.001). Buscacio et al. studied 21 eyes with preoperative corneal astigmatism of 1.06 ± 0.27 D (range 0.75 D to 1.5 D) as measured with the IOL Master 500 [25]. All the eyes received a toric IOL. Six weeks after surgery, the refractive cylinder was 0.34 ± 0.39 D (range 0.00 to 1.00). This paper had no comparison group.

Few papers dealt with “very low” preoperative astigmatism managed with toric IOLs. In the non-comparative series of Aujila and co-workers, who implanted monofocal Acrysof 1.0 D Toric IOLs in 88 eyes, the preoperative corneal astigmatism was 0.76 ± 0.18 D and the PRA was 0.26 ± 0.20 D [26]. Hao et al. studied two groups of 17 eyes implanted with the spherical or with the 1.0 D toric ReSTOR [27]. The postoperative refractive cylinder was 0.18 ± 0.17 D (toric) and 0.91 ± 0.25 D (spherical) (*P* < 0.001), but no comparison is available between groups for the preoperative corneal cylinder. Orts-Vila et al. reported about 26 eyes with preoperative corneal astigmatism of 0.62 ± 0.38 D (0.12 to 1.41), implanted with 1.0 D toric trifocal IOL [28]. They obtained a mean postoperative refractive cylinder of 0.16 ± 0.22 D (0.00 to 0.50); this study also had no comparison group.

As compared with our data, all mentioned series found lower mean PRA after 1.0 D toric IOL implantation. However, the present comparative study confirms the virtual possibility of implanting 1.0 D toric IOLs in every eye undergoing cataract surgery.

The following limitations affect the design and the conclusion of our series.

1. The number of eyes per group; although it was in line with the sample size estimation, finer statistic differences may arise from a larger study cohort.
2. The retrospective design brings with it unavoidable biases of cases’ selection, homogeneity, and comparability.
3. The use of two different models of toric and spherical IOLs and the use of a non-digital method for toric IOLs alignment.

As for now we can conclude that the use of 1.0 D toric IOLs in eyes with ≤ 1.0 D corneal astigmatism produced slight advantages in terms of PRA and uncorrected visual acuity without causing refractive damage to the considered eyes.

## Data Availability

All data and material are available from the corresponding author.
